# Improved Characterization of EV Preparations Based on Protein to Lipid Ratio and Lipid Properties

**DOI:** 10.1371/journal.pone.0121184

**Published:** 2015-03-23

**Authors:** Xabier Osteikoetxea, Andrea Balogh, Katalin Szabó-Taylor, Andrea Németh, Tamás Géza Szabó, Krisztina Pálóczi, Barbara Sódar, Ágnes Kittel, Bence György, Éva Pállinger, János Matkó, Edit Irén Buzás

**Affiliations:** 1 Department of Genetics, Cell- and Immunobiology, Semmelweis University, Budapest, Hungary; 2 Department of Immunology, Eötvös Loránd University, Budapest, Hungary; 3 Institute of Experimental Medicine, Hungarian Academy of Sciences, Budapest, Hungary; 4 Howard Hughes Medical Institute, Harvard Medical School, Boston, Massachusetts, United States of America; University of Edinburgh, UNITED KINGDOM

## Abstract

In recent years the study of extracellular vesicles has gathered much scientific and clinical interest. As the field is expanding, it is becoming clear that better methods for characterization and quantification of extracellular vesicles as well as better standards to compare studies are warranted. The goal of the present work was to find improved parameters to characterize extracellular vesicle preparations. Here we introduce a simple 96 well plate-based total lipid assay for determination of lipid content and protein to lipid ratios of extracellular vesicle preparations from various myeloid and lymphoid cell lines as well as blood plasma. These preparations included apoptotic bodies, microvesicles/microparticles, and exosomes isolated by size-based fractionation. We also investigated lipid bilayer order of extracellular vesicle subpopulations using Di-4-ANEPPDHQ lipid probe, and lipid composition using affinity reagents to clustered cholesterol (monoclonal anti-cholesterol antibody) and ganglioside GM1 (cholera toxin subunit B). We have consistently found different protein to lipid ratios characteristic for the investigated extracellular vesicle subpopulations which were substantially altered in the case of vesicular damage or protein contamination. Spectral ratiometric imaging and flow cytometric analysis also revealed marked differences between the various vesicle populations in their lipid order and their clustered membrane cholesterol and GM1 content. Our study introduces for the first time a simple and readily available lipid assay to complement the widely used protein assays in order to better characterize extracellular vesicle preparations. Besides differentiating extracellular vesicle subpopulations, the novel parameters introduced in this work (protein to lipid ratio, lipid bilayer order, and lipid composition), may prove useful for quality control of extracellular vesicle related basic and clinical studies.

## Introduction

Extracellular vesicles (EVs) comprise a heterogeneous group of lipid bilayer enclosed vesicles released by most, if not all, cells. The most extensively studied types of EVs have been classified as exosomes derived from multivesicular bodies (usually ranging from 50nm to 100nm in size [1–3], and microvesicles (often also referred to as microparticles or ectosomes) which are directly shed from the plasma membrane (mostly with sizes of 100nm to 1μm) [1,2,4]. Cells undergoing apoptosis are known to release apoptotic vesicles (up to 5 μm, [1]). The largest apoptotic vesicles are termed apoptotic bodies [2].

EVs have been found to carry and protect from degradation proteins and RNAs like mRNAs or miRNAs [5], and recent reports provide evidence also for the presence of DNA in association with EVs [6]. Consequently, different types of EVs have been implicated with roles in intercellular communication and signaling processes such as inflammation, immune suppression, antigen presentation, tumor development, as well as in the transfer of genetic information, morphogens and signaling molecules [5].

The field of EVs is emerging rapidly, and EV related biomarker and therapeutic applications make this field particularly attractive not only for basic but also for translational scientists.

Currently, many studies use total protein content determination as an integral step to quantitate and normalize the amount of EVs in preparations prior to performing downstream assays. However, an important limitation of using total protein content is that soluble proteins and protein complexes are prevalent in body fluids and culture media. Furthermore, protein aggregates can be co-purified with different EVs [7]. Additionally, membranes of EVs may rupture causing a loss in protein cargo.

Other methods used to quantitate EVs including tunable resistive pulse sensing (TRPS) and nanoparticle tracking analysis (NTA), do not provide evidence for the vesicular nature of the studied particles [8] and they require special instrumentation which may not be available in all laboratories.

In this article we propose for the first time the use of total lipid content, and protein to lipid ratio as additional parameters to total protein content and particle counts for quantifying and characterizing EV preparations. We also propose that protein to lipid ratio, lipid bilayer order (which reflects the degree of lipid packing), and selected lipid composition can also discriminate among differently sized EV subpopulations.

## Materials and Methods

### Cell lines

Jurkat (TIB-152) human T cell lymphoma and THP-1 (TIB202) human acute monocytic lymphoma cell lines were obtained from ATCC (Manassas, VA). The BV-2 murine microglia cell line was a generous gift of Prof. Rosario Donato (Università degli Studi di Perugia, Perugia, Italy) [9]. MH-S murine alveolar macrophage cell line originally obtained from ATCC was kindly provided by Dr. Dolores Solis (Madrid, Spain). The cell lines were cultured in RPMI medium containing 10% (v/v) fetal bovine serum (FBS), 2 mM glutamine, and 1% Antibiotic Antimycotic Solution (Ab/AM) (all from Sigma-Aldrich, St Louis, MO), at 37°C in 5% CO_2_/air. The cell lines were regularly tested for Mycoplasma contamination by fluorescence microscopy using DAPI staining (Molecular Probes Life Technologies, Carlsbad, CA).

### EV isolation from cell culture supernatants

Cells used for the production of EVs were grown at concentrations 0.3–1 x 10^6^/mL depending on the optimal density recommended for each cell line by ATCC. Prior to isolation, cells were washed three times with PBS, and EV production was allowed to take place for 24 hours in serum-free RPMI medium to avoid contamination of the preparations with EVs present in fetal bovine serum. Cell viability under serum free conditions for 24 hours was found to be >90–95% as confirmed by using annexin V-FITC and propidium iodide (both from BD Biosciences, San Jose, CA) staining as described before [10]. Three different EV subpopulations were isolated including apoptotic bodies (APO), microvesicles (MV), and exosomes (EXO) by the combination of gravity driven filtration and differential centrifugation. Briefly, cells were removed by centrifugation at 300g for 10 min, and then the supernatant was filtered by gravity through a 5μm filter (Millipore, Billerica, MA) and submitted to a 2,000g centrifugation for 10 min at room temperature to pellet APOs. The supernatant was next filtered by gravity through a 0.8μm filter (Millipore) and centrifuged at 12,600g for 30 min at room temperature to pellet MVs. Finally, the supernatant was ultracentrifuged in an Optima MAX-XP bench top ultracentrifuge with MLA-55 rotor (Beckman Coulter Inc., Brea, CA) at 100,000g for 70 min at 4°C to pellet EXOs. Each EV pellet was resuspended once in PBS, and recentrifuged under the same conditions that were originally used for pelleting. In the case of red blood cell derives EVs, five consecutive washes were performed.

### EV isolation from human blood plasma, activated platelets, and red blood cell concentrates

To obtain EVs from human blood plasma, we collected 30–40mL of blood from two healthy adult donors in acid-citrate dextrose tubes (ACD-A tube, Greiner Bio-One, Kremsmünster, Austria). The use of human blood samples was approved by the Scientific Ethics Committee of the Hungarian Health Scientific Council (ETT TUKEB), and human blood donors provided written informed consent. The samples were processed as described earlier [11] following the International Society on Thrombosis and Hemostasis protocol for preparation of platelet-free plasma [12,13]. Briefly, the ACD blood was centrifuged twice at 2,500g for 15 min at room temperature using a HermLe Z206A bench top centrifuge (HermLe Labortechnik GmbH, Wehingen, Germany). The supernatant was next filtered by gravity through a 0.8μm filter (Millipore) and centrifuged at 12,600g for 30 min at room temperature to pellet MVs. Finally, the supernatant was ultracentrifuged in an Optima MAX-XP bench top ultracentrifuge with MLA-55 rotor (Beckman Coulter Inc) at 100,000g for 70 min at 4°C.

For isolation of platelets, acid-citrate dextrose (ACD) plasma samples were pelleted at 400g for 15 min at room temperature to remove most of the blood cells, and then the platelet containing supernatant was diluted 4-fold in TRIS-citrate buffer. Next, the platelet-rich plasma was again centrifuged at 600g for 20 minutes at room temperature, and the pellet was resuspended in 3mL PBS. Platelets were then incubated at 37°C for an hour in the presence of 10 μM adenosine diphosphate (ADP) (Sigma-Aldrich). Next, 4mL of PBS was added to reduce the viscosity of the samples, and centrifuged at 800g for 20 min at room temperature. The supernatant was filtered by gravity through a 0.8μm filter (Millipore). After filtration, the samples were centrifuged at 20,000g for 60 minutes at room temperature to obtain the MV pellet, and then at 100,000g for 70 min at 4°C.

Red blood cell concentrates (expired by 1–7 days) were submitted for MV isolation according to the protocol described earlier [14]. Briefly, red blood cell concentrates were diluted two fold in PBS, pelleted at 1550g twice for 20 min at 20°C, and the supernatant was then centrifuged at 18,900g for 30 min in order to obtain the MV pellet.

### Tunable resistive pulse sensing (TRPS) measurements

EV preparations were submitted to TRPS analysis using a qNano instrument (IZON Science, New Zealand) as described previously [15]. First, serial dilutions were prepared from each EV preparation. Subsequently, particle numbers were counted for 5 min using 5 mbar pressure and NP200, NP400 and NP2000 nanopore membranes stretched between 45 and 47 mm. Voltage was set in between 0.1 and 0.25 V in order to achieve a stable 115nA current. Particle size histograms were recorded when root mean square noise was below 12 pA, particle rate in time was linear, and at least 500 events were counted. Calibration was performed using known concentration of beads CPC100B (mode diameter: 110nm), CPC200B (mode diameter: 203 nm) or CPC400E (mode diameter: 340nm) and CPC2000C (mode diameter: 1900nm) (all from IZON) diluted 1:1,000 in 0.2μm filtered PBS.

### Transmission electron microscopy

In order to characterize the morphology and size of the different EV preparations, pellets were fixed with 4% paraformaldehyde in 0.01M PBS for 60 min at room temperature. Following washing with PBS, the preparations were postfixed in 1% OsO_4_ (Taab, Aldermaston, Berks, UK) for 30 min. After rinsing the intact fixed pellets within the centrifugation tubes with distilled water, the pellets were dehydrated in graded ethanol, including block staining with 1% uranyl-acetate in 50% ethanol for 30 min, and were embedded in Taab 812 (Taab). Overnight polymerization of samples at 60°C was followed by sectioning, and the ultrathin sections were analyzed using a Hitachi 7100 electron microscope (Hitachi Ltd., Japan) equipped with a Megaview II (lower resolution, Soft Imaging System, Germany) digital camera.

### Flow cytometry of cells and extracellular vesicles

Cholera toxin (CTX) subunit B-Alexa Fluor647 was purchased from Life Technologies. The anti-cholesterol antibody (AC8) was produced at Eötvös Loránd University (Budapest, Hungary) [16] and conjugated to CF488A fluorophore according to the Mix-n-Stain protocol (Biotium, Sigma-Aldrich). Annexin V-FITC (in annexin binding buffer from BD Biosciences), anti-CD9 FITC and anti-CD63 PE-conjugated antibodies (all from BD Biosciences) were added to cells and EV preparations, and were incubated for 30 min at room temperature in the dark. Isotype controls (all from BD Biosciences) were used for samples stained with fluorochrome-conjugated antibodies, whereas autofluorescence was detected in the absence of either annexin V or CTX. To verify the vesicular nature of MVs and APOs, and to exclude the presence of antibody aggregates, we added Triton X-100 to 0.05% final concentration to the samples, as we described previously [7]. This step resulted in prompt disappearance of fluorescent event counts from the MV and APO gates suggesting the presence of membranous structures within these gates. Exosomes were resuspended in a total volume 50μL of PBS, and aliquots of 5μL were incubated with 5μL of 4μm aldehyde/sulfate latex beads (Life Technologies), followed by an incubation of 5 min at room temperature. Then 20μL of PBS was added, and incubation was continued for another 15 min at room temperature. Thirty μL of 2% bovine serum albumin (BSA) in PBS was added and samples were blocked for 2 hours at room temperature. Then the Eppendorf tubes were filled with PBS, and were centrifuged at 2,700g for 3 min at room temperature. The supernatant was discarded, and the pellet was resuspended in 200μL of 100mM glycine in PBS, and was incubated for 30 min at room temperature. The Eppendorf tubes were filled again with PBS, and centrifuged at 2,700g for 3 min at room temperature. Then the supernatant was discarded, and the pellet was resuspended in 100μL of PBS for staining as described above. EV preparations were analyzed by using FACSAria III and FACSCalibur flow cytometers (both from BD Biosciences). In case of APOs and MVs, instrument settings and gates were set as described earlier [12,13] using Megamix beads (BioCytex, Marseille, France) optimized with 1 μm Silica Beads Fluo-Green (Kisker, Steinfurt, Germany). Ten thousand events from equal sample volumes were counted at medium flow rate. Data were analyzed by FlowJo software (Treestar, Ashland, OR). In some experiments EVs were also stained with di-4-ANEPPDHQ (Life Technologies) at 5μM final concentration for 30 min at 37°C.

### Total protein determination of EV preparations

Total protein content of EV preparations was determined using the Micro BCA Protein Assay Reagent Kit (Thermo Scientific, Rockford, IL) following the manufacturer’s specifications and using BSA (Thermo Scientific) as a standard.

### Total lipid determination of EV preparations

To measure total lipid content of EVs, the colorimetric reaction of sulfuric acid and phosphovanilin with lipids [17] was used in a 96 well plate format sulfophosphovanilin (SPV) assay. Lipid standard solutions (2μg/μL) were prepared from menhaden fish oil and cholesterol (both from Sigma-Aldrich) in chloroform. Different volumes of the lipid standard solutions in chloroform were pipetted into Eppendorf tubes to result in 0 to 140μg of lipid per tube, and chloroform was added to each tube up to a final volume of 70μL. Chloroform (70 μL) was also pipetted onto either dry EV pellets or empty Eppendorf tubes to be used later for EV containing suspensions in PBS. The chloroform was then evaporated by incubating the tubes at 90°C for 10 min in a heater block (Techne DB-2D, Bibby Scientific Ltd, Staffordshire, UK). All steps involving chloroform handling were performed under a fume hood with lids open for all tubes. In the case when EV suspensions, as opposed to EV dry pellets were assessed, 50μL of EV suspension (with at least 10–30 μg/mL of protein depending on the EV type) was added to the empty chloroform-pretreated Eppendorf tubes and 50μL of PBS was also added to each tube containing the lipid standards. The pre-treatment with equal volumes of chloroform, and PBS in the case of EV suspensions, ensured the same background color development in all Eppendorf tubes. As a following step, 250μL of 96% sulfuric acid was added to the tubes followed by incubation with open lids at 90°C for 20 min in a heater block. Next, 220 μL of samples and standards in sulfuric acid were transferred into a 96-well polystyrene plate (Nunc, Sigma-Aldrich), and allowed to cool down to room temperature. Finally, 110μL of 0.2mg/mL vanillin in 17% phosphoric acid (both from Sigma-Aldrich) was added to each well, and the plate was incubated for 10 min at room temperature. Absorbance was measured at 540nm by an MS Reader (Multiskan MS; Labsystems, Helsinki, Finland). S1 Fig. shows a simplified schematic summary of the total lipid determination protocol.

### Assessment of interference of different compounds with lipid and protein assays

In order to assess possible interference of different substances with the total lipid and protein assays, 0μg to 20μg of tryptophan, leucine, serine, bovine serum albumin, menhaden fish oil, low molecular weight heparin, glucose (all from Sigma-Aldrich), as well as human U937 cell-derived total DNA and RNA were subjected to lipid and protein determination as described above (S2 Fig.).

Some interference by glucose was detected on the SPV assay. In the Micro BCA protein assay, both tryptophan and glucose resulted in strong colorimetric reactions. Data of n = 3 independent experiments are shown. The scale of the Y axis the amino acid interference on Micro BCA is different of the rest.

### Assessment of assay variability of lipid and protein assays

Equal concentrations of menhaden fish oil and BSA (both from Sigma-Aldrich) were subjected to total lipid and protein assays in 6 parallel technical repeats of 0, 10, 20, 30, 40, 50, 100, and 200 μg/mL concentrations in three independent experiments. Intra-assay variation was calculated as % coefficient variation (% CV) using the following formula: standard deviation (SD) of measured values/measured values x 100. Results are shown in S3A Fig. Percentage error was determined as follows: (|measured value—nominal value|/nominal value) x 100, and the results are shown in S3B Fig.

Inter-assay variation was determined as follows: average of 10μg/mL (lowest) and 200μg/mL (highest) % CVs, and were found to be 11.4 and 3.1 for the lipid and protein assays, respectively.

### Exposure of EVs to membrane damaging conditions

In order to compare intact EV preparations to those with damaged membranes, fresh EV preparations were either directly subjected to total lipid and protein determinations or were incubated overnight at 37°C before repelleting for total lipid and protein determination. In preliminary experiments incubation of MVs at 37°C overnight was found to cause vesicular damage (unpublished data).

### EV Staining with Di-4-ANEPPDHQ

In order to investigate the membrane lipid bilayer order of EV preparations, EVs were stained with the membrane probe Di-4-ANEPPDHQ (Life Technologies) at 5μM final concentration for 30 min at 37°C as described previously [18]. EV subpopulations, immobilized on poly-L-lysine (Sigma-Aldrich) coated cover slips, were analyzed by confocal microscopy (IX81 invert microscope based Fluoview500 laser scanning confocal system and software; Olympus Europe, Hamburg, Germany) at 60x magnification. Suspensions of APOs, MVs as well as 4-μm latex bead-bound EXOs were also assessed by flow cytometry. The Di-4-ANEPPDHQ probe exhibits a 60nm spectral shift between the liquid-ordered and liquid-disordered phases. Ratiometric measurement of the fluorescence intensity by confocal microscopy was performed in the 560–600nm and the >660nm emission channels, defined by appropriate band pass and cut off filters, respectively. Background-corrected fluorescence intensities of EV samples in each channel were calculated by ImageJ software (http://imagej.nih.gov/ij/). General polarization (GP) values were determined as follows: GP = (I_560–600_—I_>660_)/(I_560–600_+I_>660_). In flow cytometry, PE (585±21 nm) and PE-Texas Red (616±12 nm) filters were used to define the corresponding two emission channels and GP values were calculated in similar way.

### Statistical analysis

For data analysis we used GraphPad Prism v.4. For comparison of different parameters of EV subpopulations, we used Wilcoxon matched-pairs signed rank test. For membrane lipid order data obtained by confocal microscopy, 1-way ANOVA was used followed by Dunnetts post-hoc test. P values of less than 0.05 were considered statistically significant (* P<0.05, ** P<0.01 and *** P<0.001).

## Results

### Isolation and characterization of different EV subpopulations

**Fig 1:**
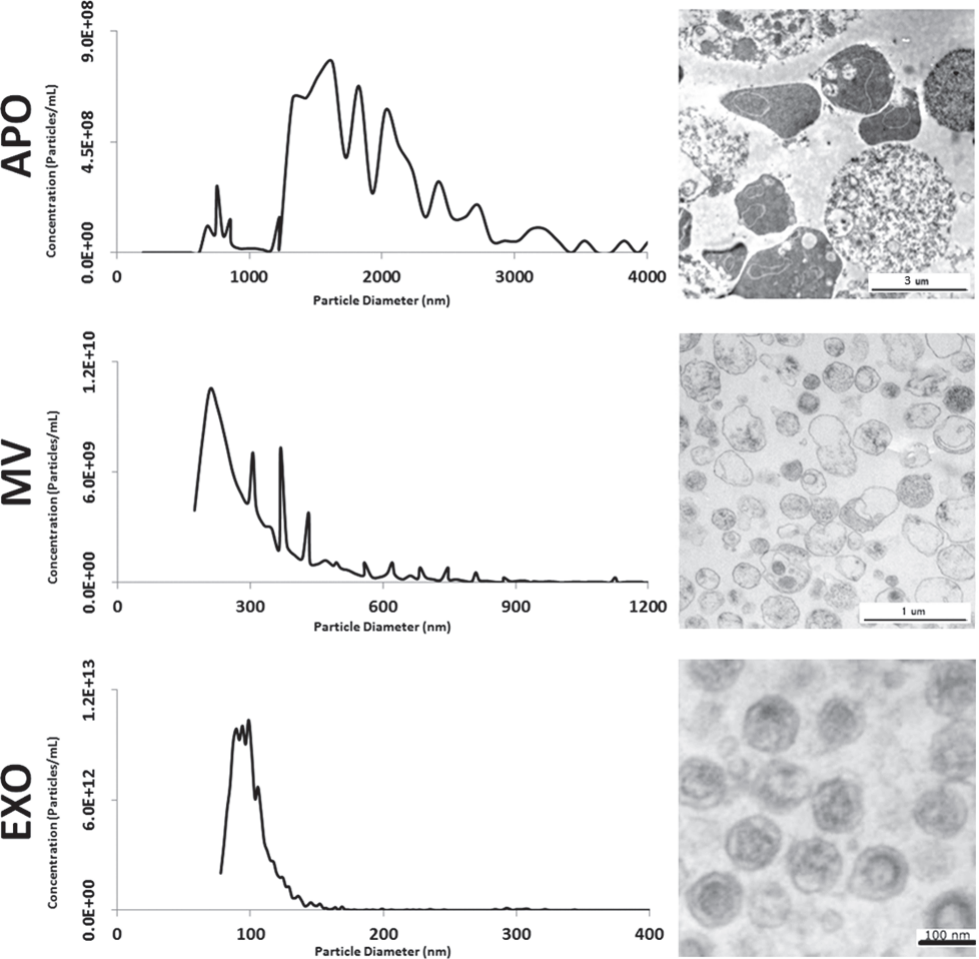
Characterization of EV subpopulation size and morphology. Apoptotic bodies (APO), microvesicles (MV) and exosomes (EXO) were isolated from conditioned tissue culture supernatant of BV-2 cells. Size distributions of the different subpopulations were assessed by tunable resistive pulse sensing (qNano) using three different membranes (NP200, NP400, and NP2000). Obtained particle concentrations were merged into single histograms for each EV subpopulation (left panels). Electron microscopic images of respective EV subpopulation pellets are shown (right panels).

EV subpopulations (APOs, MVs, and EXOs) were isolated from the supernatants of the cell lines THP-1, BV-2, MH-S, and Jurkat as described previously [10,19]. The vesicles from BV-2 cell line were characterized by tunable resistive pulse sensing (TRPS) and transmission electron microscopy. EVs from the three subpopulations were found to have different size and morphology (Fig. 1). The APO preparation predominantly contained vesicles between 1250–2500 nm in diameter (1627.05 nm mode diameter). In contrast, most vesicles in the MV preparation had approximately 200 nm diameter (208.32 nm mode diameter). The most uniform population was the one of EXOs with a peak size around 100nm (98.76 nm mode diameter). In electron microphotographs some of the APOs contained highly electron dense intravesicular content presumably corresponding to fragmented DNA. MVs were somewhat variable in shape, size and electron density, whereas EXOs had uniform size and cup shaped morphology (Fig. 1).

**Fig 2:**
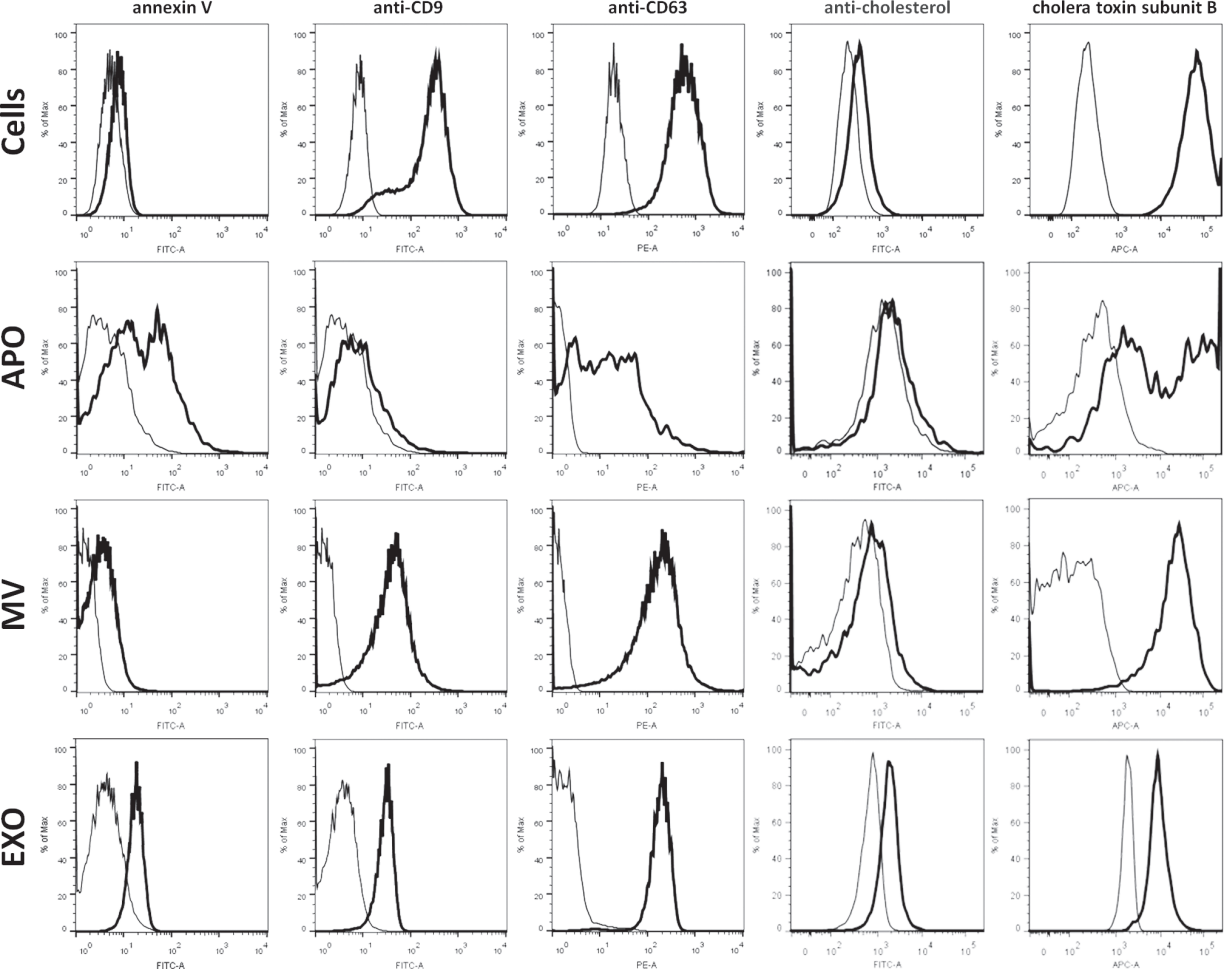
Flow cytometric characterization of EV subpopulations. EV subpopulations were isolated from BV-2 cells, and were analyzed along with the releasing cells either directly (MVs and APOs) or after coupling to latex beads (EXOs) by flow cytometry using annexin V FITC, as well as anti-CD9 FITC, anti-CD63 PE and anti-cholesterol CF488-conjugated antibodies, and Alexa Fluor647-conjugated cholera toxin (CTX) (all marked with thick black lines), and were compared to respective negative controls (thin black lines). Isotype controls were used for samples stained with fluorochrome-conjugated antibodies, whereas autofluorescence was detected in the absence of either annexin V or CTX. Images and figures are representatives of at least three independent experiments.

EVs were also characterized with flow cytometry by their staining with fluorochrome labeled annexin V, antibodies, and cholera toxin (Fig. 2). Annexin V staining that reflects phophatidyl serine externalization, was found positive for all three EV subpopulations and revealed high and low binding subpopulations within the APO fraction. While APOs exhibited minimal staining with anti-CD9, both MVs and EXOs showed strong positivity. CD63 was found to be uniformly present on both MVs and EXOs, whilst APOs showed heterogeneity in their staining (Fig. 2). The AC8 antibody that recognizes clustered cholesterol found preferentially in lipid rafts showed relatively low binding to all EV subpopulations with EXOs showing the strongest staining (Fig. 2). When staining EV subpopulations with fluorochrome-conjugated cholera toxin B (CTX) known to bind to ganglioside GM1 enriched in lipid rafts [20], we found the strongest binding to MVs while the weakest one to EXOs. Interestingly, APOs exhibited two different CTX binding subpopulations (Fig. 2).

### Determination of protein to lipid ratios in different EV populations

**Fig 3:**
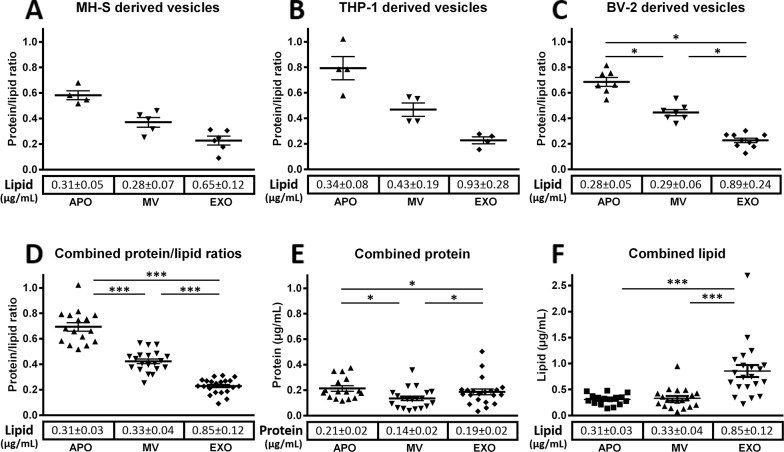
Protein to lipid ratios of EV subpopulations. Protein to lipid ratios are presented for APOs, MVs, and EXOs isolated from MH-S (A), THP-1 (B), and BV-2 (C) cell lines (data represent ≥ 3 independent experiments for each EV type from all cell lines). Combined protein to lipid ratios obtained using EVs derived from MH-S, THP-1, BV-2, Jurkat, U937 as well as from human blood plasma are shown (D) (results of ≥ 12 independent experiments each EV type). Combined protein (E) and lipid concentrations (F) for the above cell line derived and blood plasma derived EVs are also shown. Mean values are represented by horizontal lines, and standard error means (SEMs) are indicated by error bars. The mean values ± SEM of lipid and protein concentrations (μg/mL) of conditioned media are reported below each respective EV subpopulation.

Given the unmet need in the EV field for a simple and inexpensive lipid assay with the sensitivity to detect EVs, we adopted the sulfophosphovanilin (SPV) assay for total lipid determination of EV subpopulations [17,21]. In simple 96 well plate format using menhaden fish oil as a lipid standard we determined the total lipid content of EV preparations. In parallel with the total lipid determination, aliquots of the same EV preparations were also submitted to protein determination by Micro BCA assay. Total protein to total lipid ratios of EV preparations from three different cell lines (Fig. 3A-C), as well as combined protein to lipid ratios of EV subpopulations derived from all tested cell lines and human blood plasma (Fig. 3D) were found characteristic of the respective EV subpopulations. APOs were consistently characterized by the highest protein to lipid ratio followed by an intermediate ratio for MVs and the lowest ratio was in all cases found for EXOs (Fig. 3). As shown in Fig. 3E, we found differences in the combined protein concentrations of EV subpopulations of different cellular origin with MVs showing the lowest protein concentration. Interestingly, in the case of combined lipid concentrations, a striking elevation in EXOs was observed as compared to MV or APOs.

* P<0.05 and *** P<0.001

In addition, we tested different substances present in biological samples for their assay interference with the total lipid and protein determinations. We found some interference by glucose on the SPV assay (S2 Fig.), and in the Micro BCA protein assay, both tryptophan and glucose resulted in strong colorimetric reactions (S2 Fig.).

### Protein to lipid ratios in exosome-free, damaged, or contaminated EV preparations

To show the applicability of protein to lipid ratio as a quality control parameter of EV preparations, we tested EVs from different conditions.

**Fig 4:**
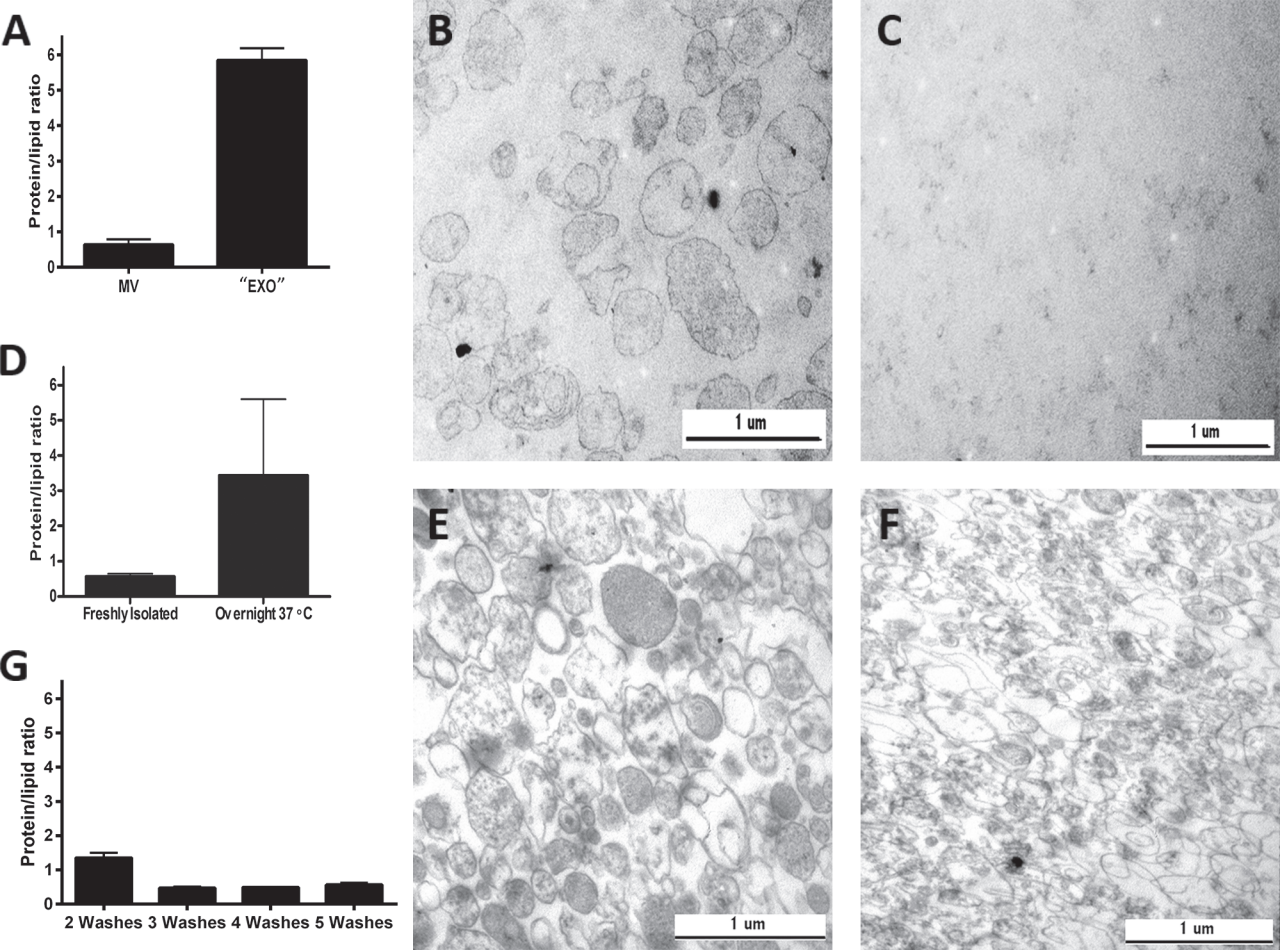
Protein to lipid ratio as a quality control parameter of EV preparations. A-C show results of 4 independent experiments where there were visible pellets for both the 20000g (MV) and 100,000g (“EXO”) preparations. While the normal protein to lipid ratio of MVs reflected a true vesicular pellet as also demonstrated by electron microscopy (B), a strongly elevated protein to lipid ratio suggested the absence of EXOs as was later confirmed by electron microscopy (C).

Firstly, platelets were incubated with adenosine diphosphate (ADP) for one hour in PBS, and the supernatant was subsequently submitted for EV isolation. The low speed (20,000g) MV pellet was characterized by the expected protein to lipid ratio (Fig. 4A) and the presence of MVs was confirmed by using transmission electron microscopy (Fig. 4B). However, the protein to lipid ratio of the high speed “EXO” (100,000g) pellet was found to be abnormally elevated (Fig. 4A). In accordance with this value, electron microscopy showed the lack of any vesicular structures in the 100,000g pellet (Fig. 4C), suggesting that under these conditions the pellet was mostly composed of proteins. This is in line with the previous finding that ADP is a poor inducer of EXO production [22].

In another experiment protein to lipid ratios obtained before and after overnight incubation at 37°C of EVs (n = 8) are shown (D). This incubation resulted in an increase in the protein to lipid ratio of MVs. As compared to the freshly isolated MVs (E), a strong deterioration of vesicular morphology was observed after overnight incubation at 37°C and repelleting of the vesicles (F) in parallel with the increased protein to lipid ratio (D). MV preparations derived from red blood cell concentrates were compared after 2, 3, 4 or 5 washes (G).

Secondly, we exposed freshly isolated MVs to overnight incubation at 37°C, a condition that we have found earlier to damage MV preparations (unpublished data). Protein to lipid ratio was determined in the low speed MV pellet before and after overnight incubation. The overnight incubated and re-pelleted MV preparation was characterized by an abnormal protein to lipid ratio compared to that of freshly isolated MVs (Fig. 4D). This was in agreement with the electron microscopy showing vesicular damage following the overnight incubation (Fig. 4E and F).

Finally MV preparations derived from red blood cell concentrates were compared after 2, 3, 4 or 5 consecutive washes by repeatedly centrifuging the samples at 18,900g for 30 min. As shown in Fig. 4G, the 3^rd^ washing step resulted in a shift from elevated protein to lipid ratio to the ratio expected for MVs suggesting the removal of contaminating proteins, and this ratio remained stable upon further washes.

### Determination of membrane lipid order in different EV subpopulations

Finally, in order to determine if membrane lipid order could be a distinguishing feature of different EV subpopulations, we stained EVs with the fluorescent polarity-sensitive lipid probe di-4-ANEPPDHQ. Using confocal microscopy and ImageJ software, we determined the general polarization (GP) values for APO, MV and EXO preparations, respectively, and found significant differences between subpopulations of EVs (1-way ANOVA, p<0.001). The GP value was calculated from the fluorescence intensities at 560–600nm and 660 nm resulting in values between −1 and +1 with higher GP values reflecting higher membrane lipid order (low liquid disordered, high liquid ordered structure).

**Fig 5:**
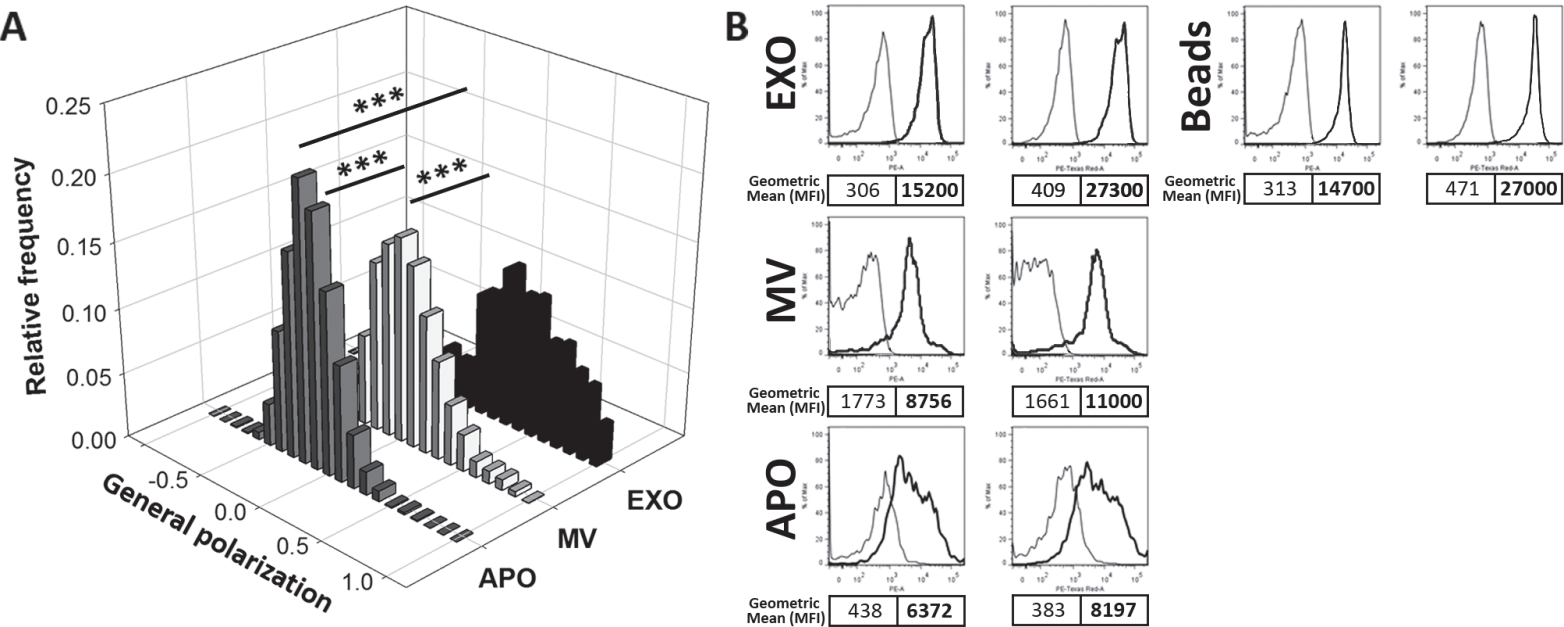
Spectral ratiometric determination of EV membrane lipid order. A shows the quantitative assessment of membrane lipid order in subpopulations of EVs secreted by BV-2 cells is shown (≥3 independent experiments each EV type). Ratiometric measurements of the fluorescent intensities at 560–600nm and at >660nm were carried out by confocal microscopy, and results are expressed as general polarization (GP) values. The higher GP value reflects a higher membrane lipid order. EXOs showed the highest order, while APOs and MVs were characterized by partially overlapping, intermediate order. B shows the flow cytometric determination of di-4-ANEPPDHQ staining of EV subpopulations secreted by BV-2 cells. Representative results of one out of n = 3 independent experiments. Left panels represent the fluorescence at 585±21 nm, while right panels represent fluorescence at 616±12 nm of unstained and stained vesicles and beads (thin and thick lines, respectively). Geometric mean fluorescent intensities (MFI) of unstained and stained APOs, MVs, EXOs coupled to latex beads, and beads without EXOs are shown (regular and bold text, respectively).

APOs and MVs were characterized by intermediate and partially overlapping lipid orders with GP values of −0.15 ± 0.15 and 0.00 ± 0.21 (median ± SEM), respectively. In contrast, EXOs displayed a GP value of 0.36 ± 0.29 (median ± SEM), reflecting a remarkably higher degree of lipid order than either MVs or APOs (Fig. 5A). The observed GP values were also assessed by flow cytometry and the results were close to those of the confocal microscope measurement. The GP values measured by flow cytometry were −0.14, −0.14, and 0.25 for APOs, MVs, and EXOs, respectively (Fig. 5B). The differences between the GP values measured by confocal microscopy and flow cytometry could be ascribed to the small differences in the available filters in each instrument (560–600nm and >660 nm in the confocal microscope as opposed to 585±21 nm and 616±12 in the flow cytometer). However, the pattern of higher order in EXOs was observed by both instruments.

## Discussion

In the past 15 years EV research emerged as a novel field of cell biology. Understanding of the roles of EVs has profoundly impacted our understanding of intercellular communication, tumor and stem cell biology, inflammation, virology, circulating extracellular RNA and DNA research among other fields [1,4,5,23].

In the meantime, different methods have been adopted to quantify EVs. However, currently used assays either do not discriminate between vesicular or non-vesicular particles (such as NTA and TRPS), or solely quantify EVs in a given preparation based on the total protein content. Since protein aggregates have been shown to be co-purified with EVs [7], and in some samples soluble proteins and protein aggregates are more abundant than lipid bilayer enclosed EVs, such approaches are error-prone. We hypothesized that as an alternative to protein-based EV quantification, simultaneous determination of both the protein and lipid content may lead to a better quantification of the EVs in a sample. In spite of the large body of published data on EVs, virtually none of the studies have used an assay to determine total lipids in EV preparations besides determining the protein content. Thus, there has been an unmet need in the field for a simple lipid assay to detect EVs. Furthermore, there have been no universally accepted molecular markers to distinguish among the subpopulations of EVs.

This study was undertaken to establish a simple lipid assay for EVs and to find good quality control parameters to characterize EV preparations that might be utilized both in basic research and in clinical laboratory settings.

For the first time we used and optimized the SPV total lipid assay for EV studies and combined this lipid assay with the conventional Micro BCA protein determination that is used widely in the EV field. This total lipid assay is simple and fast requiring only 30 min of incubation, and 0.5μg protein containing EVs either in dry pellet or in up to 50μL volume. Furthermore, we showed if used under the optimized conditions described in this paper, the SPV assay sensitivity is adequate for EV studies and compares favorably to Micro BCA in terms of accuracy, although shows slightly higher intra and inter-assay variabilities. However, at concentrations >50 μg/mL lipid for SPV or protein for Micro BCA, both assays show low variability and good accuracy (S3 Fig.). Using these two assays, we introduced protein to lipid ratio as a novel parameter to characterize EV preparations (EXOs, MVs, and APOs). As expected, we found increasing protein to lipid ratios for EV subpopulations of increasing diameter. Our data are in line with results of an earlier study that determined protein and lipid composition of platelet microparticle size fractions using mass spectrometry and a detection of lipid phosphorous with the method of Bartlett [24]. We found that the protein to lipid values depended on the lipid standard used in the assay. Although the reference lipids (such as menhaden fish oil) that we used in this study may not exactly reflect the complex composition of EV lipid membranes, they are produced under standardized conditions, they are inexpensive and they are commercially available for the broad scientific community. More sophisticated lipid standards may be further developed as the lipid composition of EVs will be better understood. Regardless of the method used, if adhering to the same lipid standard consistently throughout the experiments, protein to lipid ratio determination may prove very useful for quality control of EV preparations. In addition to the SPV method introduced in this study, more sophisticated methods such as infrared spectroscopy could be alternatively used for the determination of protein and lipid content of EVs. However, the requirement for special equipment may prevent the use of infrared spectroscopy in many laboratories. Furthermore, cholesterol as well as other sterols do not have many useful infrared features by which to distinguish them from other molecules. The vibrational bands due to C–C or C-H bonds, which are commonly used to measure sterols with infrared spectroscopy, are not only very weak but are also shared by most biological molecules including proteins [25]. Its reduced cholesterol sensitivity may thus, limit the wide use of infrared spectroscopy for EV studies.

Besides EVs, we also determined the protein to lipid ratio of cells releasing EVs. We found that, as compared to the different EV subpopulations, cells showed substantially higher variation of protein to lipid ratios probably reflecting differences in cell types and in cell cycle phases (data not shown). Lipid sorting mechanisms (such as preferential sorting of cholesterol into EXOs) during the generation of different EV subpopulations have been reported [26, 27]. Such sorting process may result in the enrichment of given lipids in different EV subpopulations which may contribute to the relatively low variation of protein to lipid ratios of the same EV subpopulation compared to the producing cells.

Next, in an attempt to find novel parameters to characterize EV subpopulations, using flow cytometry we stained our EVs with an anti-cholesterol antibody, and found strongest staining for EXOs. Furthermore, we found staining of all EV subpopulations with CTX reflecting the presence of GM1 gangliosides in all EV subpopulations. GM1 gangliosides have been previously detected in EXOs [28, 29]. Here we found that GM1 was not only present in EXOs but also especially enriched in MVs and differentiated two subpopulations of APOs. Intriguingly, heterogeneity within APOs was not only suggested by bimodal CTX binding, but also by the annexin V and anti-CD63 staining. Transmission electron microscopy also revealed the presence of highly electron dense, possibly fragmented DNA containing APOs and less electron dense and more granular APOs possibly corresponding to the recently described different subpopulations of APOs [30].

Our flow cytometry data are in accordance with earlier findings that EXOs are particularly enriched in cholesterol and GM1 gangliosides [26,31].

The spectral ratiometric approach of this study provides evidence that EV subpopulations can be distinguished based on the difference in their membrane lipid order. This parameter reflects the degree of lipid packing and is one of the most important biophysical parameters of membranes [18]. In low ordered membrane domains the probability of protein-protein interactions is decreased. Therefore membrane lipid order can impact signaling pathways. This is strongly supported by the findings that high membrane lipid order is typically found at the immunological synapse, sites of cell adhesion, viral entry and budding as well as in exosomes (the latter described for the first time in this work). Our data suggest that EXOs are characterized by highest membrane lipid order, while APOs and MVs showed low to intermediate, partially overlapping lipid order reflecting important differences in membrane lipid composition. A high degree of membrane lipid order and the relatively high content of cholesterol in EXOs may be important factors explaining their distinguished role in intercellular signaling.

There is an ongoing debate in the EV field about the classification of different subpopulations of EVs. Without universally accepted molecular markers reflecting different biogenic origin of EV subpopulations, many studies simply classify EVs based on their size. Such a size-based classification could be challenged by observations that exosome-sized EVs can also be shed from the plasma membrane [32]. In an attempt to assess whether this kind of observations represent exceptions to the rule or if size-based classification is arbitrary, we looked beyond size, and compared lipid properties and protein to lipid ratios in EVs fractionated by size. We found differential binding of an anti-cholesterol antibody and CTX to different sized populations. Additionally, our data suggest the decreasing protein to lipid ratio of decreasing sized EV subpopulations is not solely dependent on a diminished surface area to volume ratio. S1 Table shows that instead we found a smaller increase in the protein to lipid ratios than what was expected based on geometry (surface area to volume ratio). This may either represent different cargo packing densities or different molecular composition of the different EV subtypes.

Our observations suggest that simple size-based classification enables distinguishing EVs with different membrane lipid properties. Until selective molecular markers become available, established protein to lipid ratios may serve as suitable parameters to characterize different EV subpopulations. In addition, protein to lipid ratio may also prove useful for routine quality control of EV preparations.

## Supporting Information

S1 Fig A simplified schematic summary of the total lipid determination protocol. (TIF)

S2 Fig Interference with the 96 well plate Micro BCA total protein and SPV total lipid determination assays. (TIF)

S3 Fig Working concentration ranges and coefficients of variations of the Micro BCA and SPV assays. S3A Fig. shows precision profile as defined by Carrol (2003) indicating the working concentration range by percentage coefficient intra-assay variation (%CV) plotted against concentration for both SPV total lipid assay and Micro BCA total protein assay. The suggested working range (gray box) is defined as the range of concentrations for which the coefficient of variation is <20%. S3B Fig. shows the intra-assay percentage error (% Error) for the same assays reflecting higher accuracy of the SPV measurements. The working range as suggested in S3A Fig. is also indicated by a gray box. The scales of the Y axis are not the same. n = 3, 6 technical parallels for each concentration.
(TIF)

S1 Table Calculated volumes to surface area ratios for the size ranges of different EV subpopulations in comparison with the observed protein to lipid ratios. (TIF)
