# Supplementary figures and images for: Improved Characterization of EV Preparations Based on Protein to Lipid Ratio and Lipid Properties
Source: PLoS One. 2015 Mar 23;10(3):e0121184. doi: 10.1371/journal.pone.0121184 (PMC4370721; doi:10.1371/journal.pone.0121184)

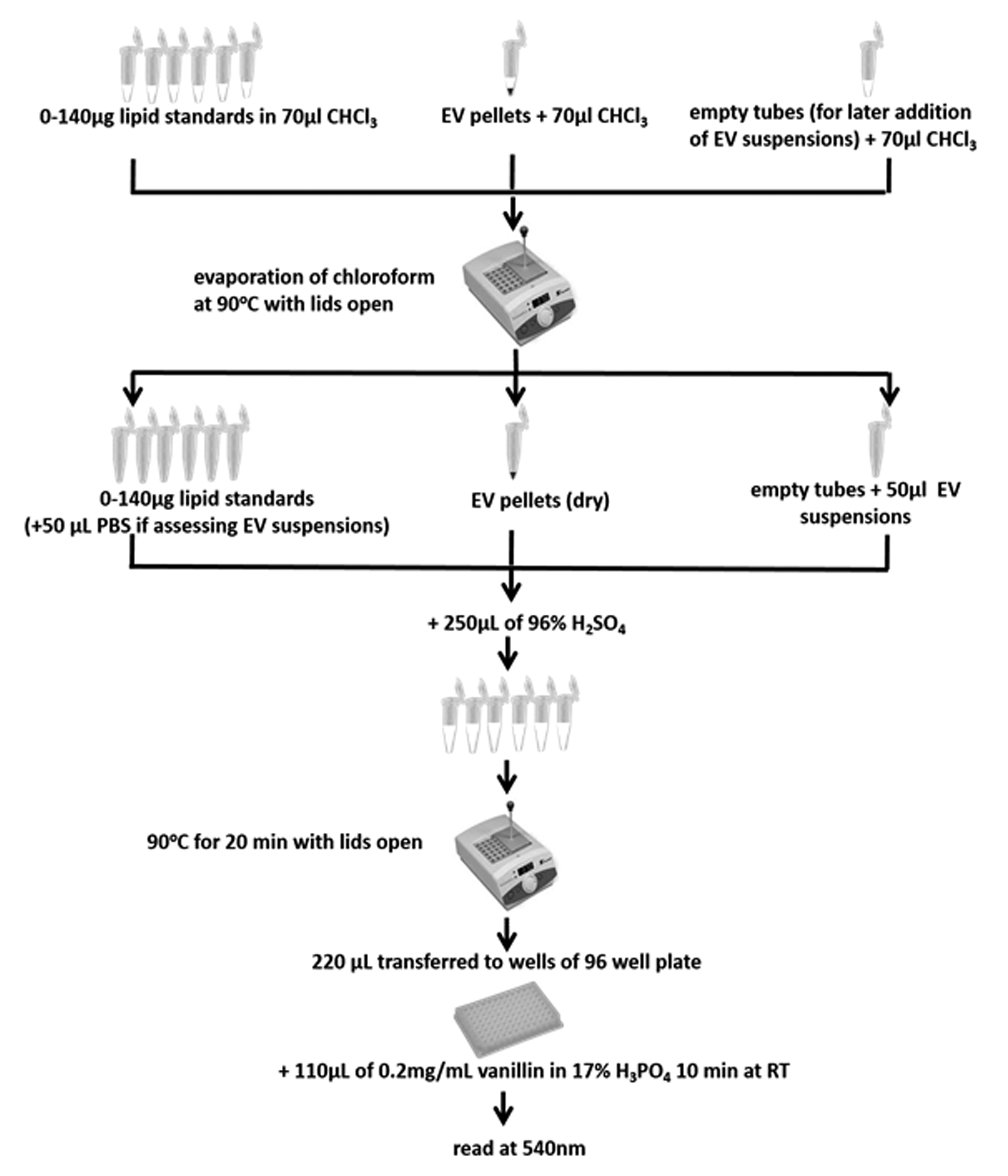

Supplement: S1 Fig — (TIF) [file pone.0121184.s001.tif]

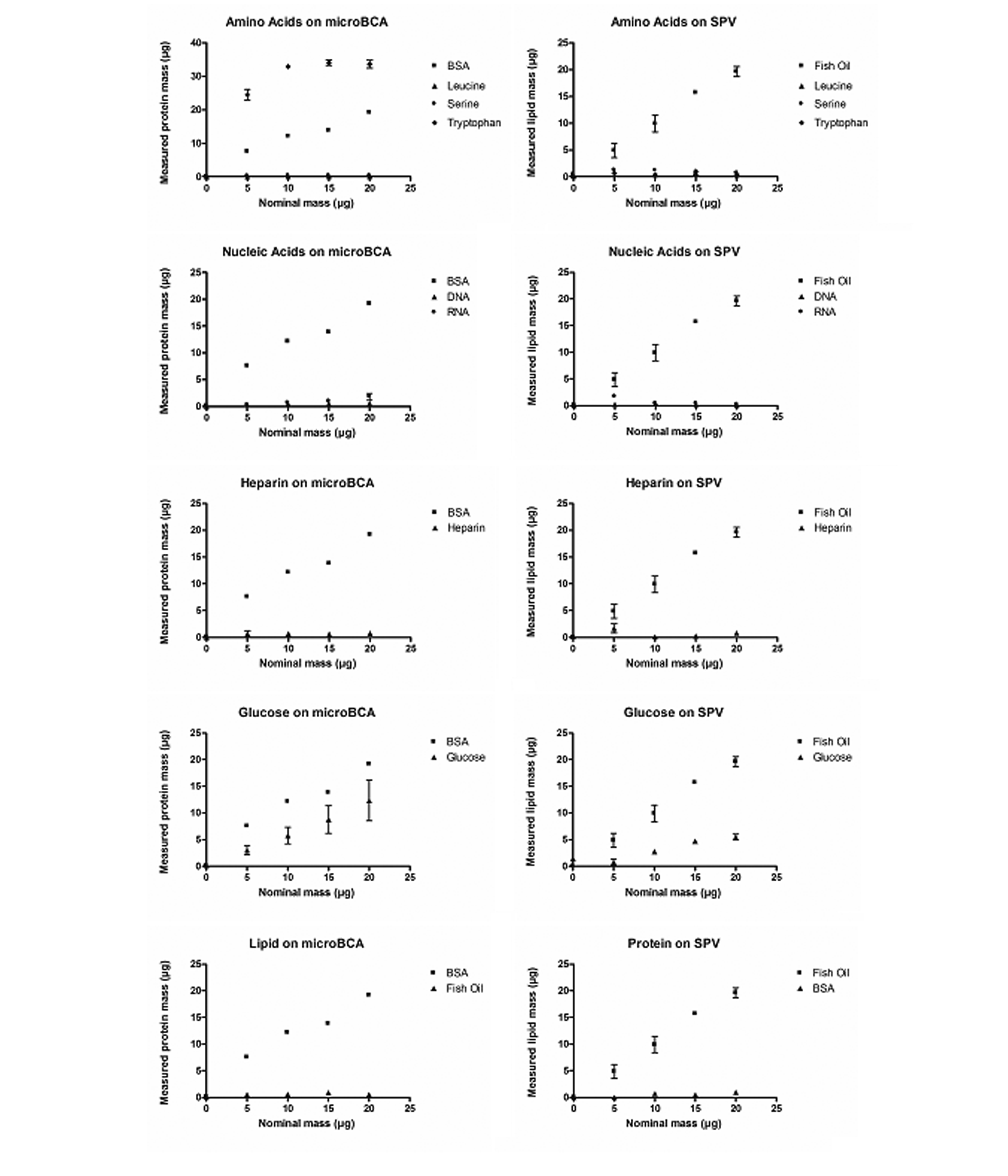

Supplement: S2 Fig — (TIF) [file pone.0121184.s002.tif]

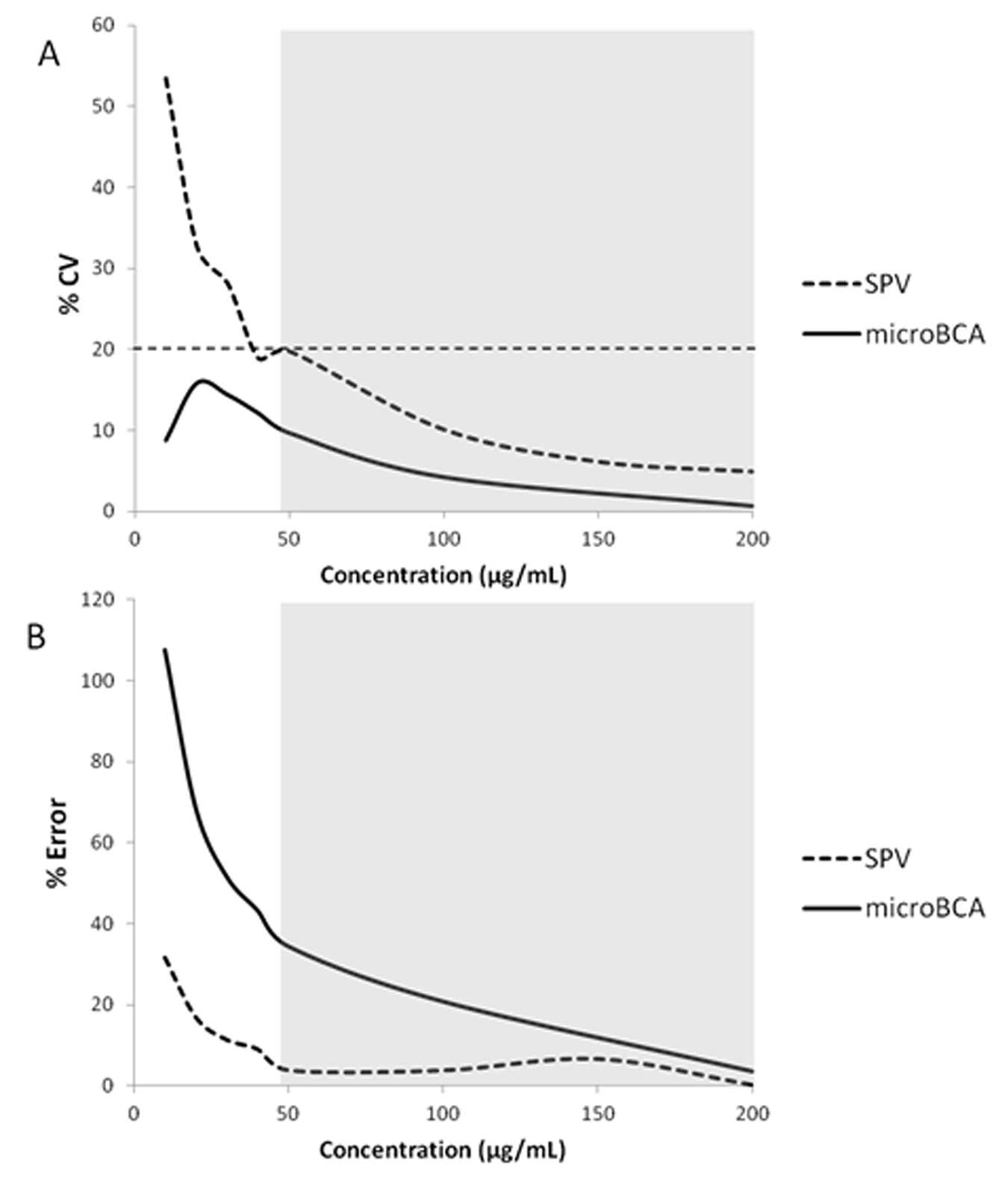

Supplement: S3 Fig — S3A Fig. shows precision profile as defined by Carrol (2003) indicating the working concentration range by percentage coefficient intra-assay variation (%CV) plotted against concentration for both SPV total lipid assay and Micro BCA total protein assay. The suggested working range (gray box) is defined as the range of concentrations for which the coefficient of variation is <20%. S3B Fig. shows the intra-assay percentage error (% Error) for the same assays reflecting higher accuracy of the SPV measurements. The working range as suggested in S3A Fig. is also indicated by a gray box. The scales of the Y axis are not the same. n = 3, 6 technical parallels for each concentration. (TIF) [file pone.0121184.s003.tif]

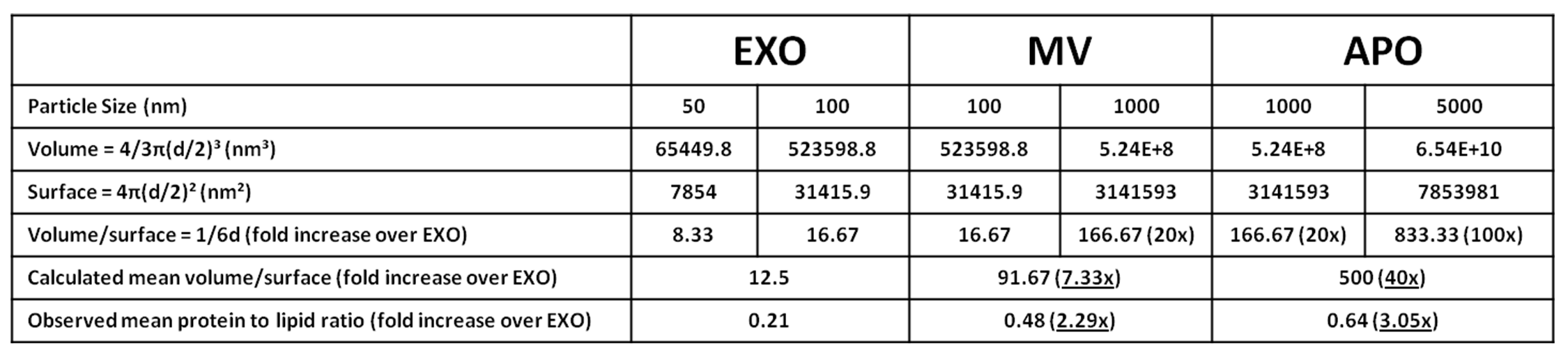

Supplement: S1 Table — (TIF) [file pone.0121184.s004.tif]
